# Is There a Biological Basis for Treatment of Fibrodysplasia Ossificans Progressiva with Rosiglitazone? Potential Benefits and Undesired Effects

**DOI:** 10.1155/2010/541927

**Published:** 2010-06-16

**Authors:** Renata Bocciardi, Roberto Ravazzolo

**Affiliations:** ^1^Molecular Genetics Unit, G. Gaslini Institute, 16147 Genova, Italy; ^2^Department of Pediatrics, Center of Excellence for Biomedical Research (CEBR), University of Genova, 16147 Genova, Italy

## Abstract

Thiazolidinediones (TZDs), among which Rosiglitazone, are known agonists of the peroxisome-proliferator-activated receptor *γ* (PPAR*γ*) commonly used for treatment of hyperglycemia. A recently published article describing a case report on a patient affected by Fibrodysplasia Ossificans Progressiva (FOP) treated with Rosiglitazone has prompted interest for careful analysis of the rational basis of such treatment. This article reviews the effects of PPAR*γ* agonists in relationship with various pathogenic steps that occur during the course of FOP by reviewing the particularly rich literature on the effects of Rosiglitazone, to underscore their relevance to FOP and to consider possible adverse effects.

## 1. Fibrodysplasia Ossificans Progressiva

Fibrodysplasia Ossificans Progressiva (FOP) (MIM 135100) is a rare and extremely disabling disorder, characterized by congenital malformation of the great toes and progressive heterotopic ossification [1, 2].

Heterotopic ossification generally starts in the infancy and continues in an episodic and progressive way. Typically, the disorder presents with alternate quiescent periods, that sometimes last also years, and acute phases (flareups) through which ectopic bone formation at the level of axial and appendicular skeleton occurs and causes progressive and severe joint ankyloses and formation of an ectopic “second skeleton” [3].

The molecular defect causing the disorder is a gain of function mutation in the *ACVR1/ALK2* gene, that encodes a Type I receptor for bone morphogenetic proteins (BMPs) [4]. The large majority of patients carry a heterozygous recurrent mutation, c.617G>A, causing an R206H substitution. This mutation affects the protein Glycine Serine-rich (GS) domain, which is highly conserved across species and has regulatory function [4]. Much less frequently, FOP is associated with substitution of other residues in the same GS domain or in the kinase domain [5, 6]. The functional consequence of the *ACVR1*/*ALK2* mutation, accurately characterized by *in vitro* studies for the most frequently occurring R206H mutation, is anomalous ligand-independent activation and hyper-response to BMP ligands of the SMAD-dependent signaling pathway [7–9]. Thus, these results are consistent with the mechanism of ectopic ossification caused by a gain of function mutation in the *ACVR1*/*ALK2* gene.

The quite mild congenital malformation in the great toes can be interpreted in the context of the complex interplay of developmental pathways, including the BMP pathway, controlling development of limb elements. The most critical and still poorly understood point in the pathogenic mechanism is the type of stimuli that trigger flareup episodes and subsequent ossification. 

Flareups seem to be induced by trauma, medical or surgical or dental interventions, intramuscular injections, and infections. However, in some cases flareups start with no apparent stimulus. The disorder is highly variable in term of onset, frequency, and severity of ectopic ossification episodes [3]. 

Pathological findings described after analysis of bioptic specimens from misdiagnosed patients have highlighted two important points: the phases of tissue composition at the sites of flareup and the type of bone produced in ectopic lesions [10–14].

Bone formation is preceded by the appearance of bumps that very often cause misdiagnosis by erroneous attribution of the initial lesion to cancer or other disorders like aggressive juvenile fibromatosis or lymphoedema [3]. The histological analysis [10–14] shows characteristics of inflamed tissue with perivascular infiltration of B and T lymphocytes extending towards the surrounding muscular tissue and inducing its necrotic process. This initial phase is followed by an intense fibroproliferative reaction, vascularisation, and angiogenesis. After the fibroproliferative phase ectopic bone formation takes place through a normal endochondral intermediate phase. Mast cell infiltration is observed in all phases and is particularly abundant in the initial phases, further supporting the inflammatory nature of the initial stimulus [13, 14]. 

FOP ectopic bone appears as a normal endochondrally formed lamellar bone with normal bone marrow elements. Radiological and scintigraphic characterization of ectopic bone in 47 FOP patients showed characteristics of resistance, stress response, and remodeling comparable to normal bone in unaffected people [15].

Thus, an inflammatory stimulus that in some way reaches a mutated receptor and stimulates a hyper-responsive signaling pathway are essential elements in the pathogenic process. Many efforts and expectations are directed to find approaches to interfere with the most critical steps of the pathogenic mechanism that could beneficially affect patients' quality of life.

Very recently an article that describes successful results of treatment of an FOP patient with Rosiglitazone has been published [16], which stimulates an in depth analysis of possible effects of such treatment in relationship with various pathogenic steps that occur during the course of FOP.

The aim of this article was to review a particularly rich literature on the effects of Rosiglitazone and try to underscore their relevance to FOP.

## 2. Rosiglitazone

Rosiglitazone (ROSI) belongs to the class of Thiazolidinediones (TZDs) and has been approved as drug for treatment of hyperglycemia [17]. Other TZD molecules are Troglitazone, first approved drug in this class that was withdrawn from the market because of severe drug-induced liver failure, and Pioglitazone, presently available for therapeutic uses as Rosiglitazone. 

Rosiglitazone is mainly used as an orally active hypoglycemic agent for the treatment of noninsulin-dependent diabetes mellitus or Type 2 Diabetes (T2D). 

### 2.1. Mechanism of Action: ROSI as an Antidiabetic Drug

One of the characteristics of T2D is impaired response to normal circulating insulin levels at target tissues (muscle, liver, adipose tissue). Pancreatic beta cells partly compensate this resistance by increasing their own mass and/or efficiency of insulin secretion, however, when this compensatory ability is insufficient, hyperglycemia occurs. ROSI acts by the highly selective activation of the Peroxisome-Proliferator-Activated Receptor gamma (PPAR-*γ*), a ligand dependent transcription factor that belongs to the family of nuclear hormone receptors [17, 18]. PPAR-*γ* is expressed in tissues that are key targets for insulin action such as adipose tissue, skeletal muscle, and liver. PPAR-*γ* activation positively regulates transcription of insulin responsive genes involved in glucose control of synthesis, transport, and utilization, thus contributing to restore sensitivity to insulin at target organs and to glycemia control.

Because of its actions at different levels, adverse effects such as appearance or worsening of cardiovascular events, overweight, edema, and increased risk of fractures are observed.

## 3. Peroxisome-Proliferator-Activated Receptor Gamma (PPAR-*γ*)

PPAR-*γ*, the transcription factor for which ROSI is a potent and selective agonist, is involved in several different physiological and pathological processes such as differentiation, inflammation, aging, obesity, infertility, and cancer [19–21]. In particular it is essential for adipogenesis, glucose homeostasis, and control of inflammation.

Three PPAR isotypes, with different expression patterns, are known.

- PPAR-*α* is expressed in tissues with high catalytic activity of fatty acids as liver, skeleton, cardiac muscle, renal cortex.
- PPAR-*δ* has a wide expression pattern in particular in tissues where it exerts control of fatty acid oxidation as skin, brain, adipose tissue, muscle.
- PPAR-*γ*, the most extensively studied, is highly expressed in adipose tissue where it acts as the central molecule for adipocytic differentation, in pancreatic beta cells, vascular endothelial cells, and different cells involved in immune response as monocytes, macrophages, dendritic cells. PPAR-*γ* isoforms, differing for alternative promoters, and mRNA processing, are reported [21].

The different PPAR molecules share a four domain structural oganization [19, 21]. The A/B NH2 terminal domain, also named AF1 domain, is responsible for ligand-independent transcriptional activity. The cDNA binding domain is necessary for recognition of consensus sequences in target gene regulatory regions, called PPAR response elements (PPREs). The D domain, is essential for modulation of DNA binding thanks to binding of specific cofactors. A transcriptional activation domain, named AF2, has the role of both binding the ligand and mediating heterodimerizaton with the obligate partner nuclear receptor for 9-cis retinoic acid (RXR) [19, 21].

PPAR-*γ* transcriptional activity, either positive (transactivating) or negative (transrepressing), is modulated both by the interaction with a ligand and by posttranslational modifications. Interaction with the ligand induces heterodimerization with RXR and DNA binding. The simultaneous release of repressor cofactors and recruitment of coactivators also occur. PPAR-*γ* can interact with different endogenous ligands, such as fatty acid oxidized metabolites and 15-deoxi-D-prostaglandine [22, 23], or exogenous ligands as flavonoids, linoleic or eicopentanoic or decoesanoic acids present in the diet, and synthetic molecules as the TZD class molecules ROSI, Pioglitazone, and Troglitazone [24].

PPAR-*γ* phosphorylation at specific residues, Ser 82/112 and Ser 84 in the AF1 domain, by different kinases (ERK, JNF-MAPK, AMPK), has a negative effect on transcriptional activation both ligand-dependent or independent [19, 21]. 

Another mechanism of transcriptional repression is due to ligand-dependent sumoylation at the ligand-interaction domain of PPAR-*γ*. Sumoylation causes formation of a complex including PPAR-*γ*, a nuclear receptor corepressor (NcoR) and histone deacetylase-3 (HDAC3). This complex can be localized at PPREs in promoters of proinflammatory genes related to the NF*κ*B that are therefore repressed [25]. 

### 3.1. PPAR-*γ* as an Anti-Inflammatory Molecule

PPAR-*γ* is normally expressed in antigen presenting cells in the immune system, as monocytes, macrophages, and dendritic cells [26–28]. Treatment of these cells with PPAR-*γ* agonists such as TZDs causes suppression of inflammatory cytokines production [29, 30]. Accordingly, analysis of gene expression profile in murine macrophages showed that PPAR-*γ* exerts a general repressive action on a wide range of target genes inducible by LPS or Interferon gamma (IFN*γ*) [31].

Generation of PPAR-*γ* conditional knock out mice in macrophages further underscored its importance in control of inflammation and oxidative metabolism [32].

Macrophages represent a first line defense against pathogens and, in response to microenvironment stimuli, may become activated and differentiate by different ways. The classic activation is induced by Interferon-*γ* and promotes a proinflammatory response to elimininate pathogens [22]. An alternative activation pathway induced by cytokines like IL-4 and IL-13, leads to a different phenotype with an important role in control and limitation of the inflammatory response and tissue damage repair [22]. PPAR-*γ* is involved in such alternative maturation process and therefore plays an important anti-inflammatory role. 

Other evidences underscore the anti-inflammatory role of PPAR-*γ*. Animal models of inflammatory and autoimmune disorders as asthma, rheumatoid arthritis, multiple sclerosis, and type 2 diabetes associated nephropathy [24, 25, 33], has highlighted the efficacy of treatment with PPAR-*γ* agonists in improving several signs associated with these diseases. As an example, Pioglitazone results equally efficient as common treatments with corticosteroids for murine models of asthma [33]. Pioglitazone and ROSI reduce bone erosion secondary to inflammation in rat models of rheumatoid arthritis [34]. These studies have provided the proof of principle for the use of PPAR-*γ* agonists in clinical trials for treatment of multiple sclerosis and ulcerative colitis and for already approved trials for treatment of patients with rheumatoid arthritis and asthma [35–37].

Together with the above evidences, a common PPAR-*γ* (Pro12Ala) nonsynonymous polymorphism that causes substitution of the conserved Pro12 residue in the ligand-independent DNA binding domain and other less common variants have been evaluated in association studies and also investigated by functional studies [38]. The variant protein shows differential activation of target genes with respect to the wild type protein. Moreover a protective role of the Pro12Ala variant was reported in (a) patients with multiple sclerosis who show a disease later onset [39]; (b) male patients with coronary artery disease who show less severe and less generalized atherosclerosis with reduced morbidity and mortality of cardiovascular events [40]; (c) patients with type 2 diabetes who show higher insulin sensitivity [40].

Besides mediating the antidiabetic and anti-inflammatory effects of TZD agonists, PPAR-*γ* was also reported as a target of 5-aminosalicylic acid (5-ASA) anti-inflammatory effects in an animal model of inflammatory bowel disease [41].

### 3.2. PPAR-*γ*: Adipogenesis versus Osteogenesis

Osteoblasts and adipocytes share common progenitors, mesenchymal stem cells.

PPAR-*γ* expression is early induced by different key factors in the adipogenesis pathway and is considered the master transcriptional regulator of adipogenesis [42]. Fibroblastic cell lines start adipogenic differentiation after transfer of PPAR-*γ* cDNA and murine myoblasts transdifferentiate to mature adipocytes [43]. Phenotypic conversion of these cells to adipocytes is accompanied by induction of genes responsible for metabolism and secretion of fatty acids, tryglyceride synthesis, and transport of long chain fatty acids. 

Generation of PPAR-*γ* knock out mouse models were critical for defining the effects of changes in PPAR-*γ* levels *in vivo*.

Null homozygous mice showed lethality during embryo development at 10.5–11.5 dpc because of placental dysfunction [43]. Interestingly, analysis of mice heterozygous for the null allele [43] which showed normal development and growth on a standard diet, also showed protection from obesity and insulin resistance under high fat content diet, that were abrogated by treatment with PPAR-*γ* agonists. The protective state in heterozygotes was associated with a nearly 2-fold higher leptin expression under high fat diet. Studies on these mice to examine the effect of PPAR-*γ* deficiency in bone metabolism [44] showed that Embryonal Stem Cells from homozygous nulls were unable to undergo adipogenesis, while osteogenesis was spontaneously occurring in the absence of osteogenic inducers. Treatment of the same cells with PPAR-*γ* restored adipogenesis and decreased osteogenesis indicating a coordinated opposite regulation of the two processes by PPAR-*γ*. The observation of heterozygous mice confirmed an apparent normal growth under standard diet, including the length of trunk and long bones. However, radiological and densitometric analyses show a 40% increase of trabecular bone mass and concomitant reduction of marrow adipocyte number in long bones and vertebrae. Accordingly, primary bone marrow cells from heterozygotes showed increased osteoblastogenesis in the absence of differentiating agents, with upregulation of key molecules for osteoblast differentiation, *Runx2, osterix, and LRP5*. Although the switching mechanism between the adipogenic and osteogenic differentiation pathways from common progenitors was not fully clarified, these studies clearly indicated that PPAR-*γ* acts as a potent suppressor of commitment and differentiation to the osteoblastic lineage. Another contribution by the study of PPAR-*γ* deficient mice [44] was the finding that PPAR-*γ* signaling does not seem to be involved in osteopenia caused by estrogen deficiency while seemed to be involved in age-related bone loss possibly due to gradual increased adipogenic differentiation at the expense of osteoblastogenesis.

Another mouse model, the PPAR-*γ* ^hyp/hyp^, in which a hypomorphic mutation at the PPAR-*γ* locus causes absence of PPAR-*γ* expression in white adipose tissue (WAT), has highlighted the contribution of PPAR-*γ* to bone homeostasis [45]. The homozygous mice, which are severely lipodystrophic, showed increase of bone mineral density, bone area and bone content, and bone trabecular thickness, demonstrating that the decrease in PPAR-*γ* activity in fat enhances bone formation. Increased osteoblast activity was consistent with increased expression of osteoblast-specific transcription factors and markers. In association to lipodystrophy, strong reduction of leptin levels was observed, known to enhance bone formation in mice and humans [46]. These mice also exhibited increased osteoclast activity (see following section) and extramedullary hematopoiesis probably explained by bone marrow space limitation.

### 3.3. PPAR-*γ* and Osteoclastogenesis

Considering the observed reduced bone mass after treatment with TZD PPAR-*γ* agonists, both in animal models and in clinical studies in diabetic human patients, the question whether these drugs, besides affecting osteoblastogenesis, also affect osteoclastogenesis also arises. Osteoclasts are multinucleated cells derived from hematopoietic precursors, contrary to osteoblasts that derive from mesenchymal stem cells. 

As mentioned above, PPAR-*γ* ^hyp/hyp  ^ mice showed increased osteoclast activity documented by increased expression of genes typical of the osteoclastic lineage, tartrate-resistant acid phosphatase (*Trap*) and cathepsin K (*CathK*) [45].

On the other hand, mice generated with conditional knock out of PPAR-*γ* in hematopoietic and endothelial cells showed evident splenomegaly with megakaryocyte accumulation, extramedullary hematopoiesis, pale bones, increased bone volume with reduced marrow cavity [47]. After hematopoiesis established in spleen in the place of bone marrow, the mice showed normal hematopoiesis with normal activation and expression of specific genes and markers. Reduction of marrow cavity and increase of bone volume suggested a defective bone remodeling because of defect of osteoclast function. Osteoclasts were reduced in number while osteoblasts were normally present. Early osteoclast markers, such as tartrate-resistant acid phosphatase (*TRAP*), calcitonin receptor (*Calcr*), carbonic anhydrase 2 (*Car2*), cathepsin K (*Ctsk*), resulted defective due to lack of *RANKL* direct transcriptional activation by PPAR-*γ* and consequent defect of an essential pathway for differentiation of precursor cells to mature osteoclasts [47]. Treatment with ROSI of progenitor cells from wild type mice led to stimulation of osteoclast production, that did not take place when cells were taken from PPAR-*γ* defective mice. The defect of the RANKL pathway appeared associated with signaling through *c-fos*, since the differentiation block could be released when the mutant cells were infected with a viral vector expressing exogenous *c-fos* cDNA. The extramedullary hematopoiesis in the mutant mice could be corrected by transplantation of bone marrow from normal mice, whereas the reverse transplantation of bone marrow from PPAR-*γ* mutant mice to normal mice caused appearance of extramedullary hematopoiesis.

In summary, although with some contradiction in published data (increased osteoclast activity in [45] versus defective osteoclast activity in [47]), several evidences support the involvement of PPAR-*γ* in osteoclast differentiation and bone remodeling.

### 3.4. Rosiglitazone and Bone: Effect of Treatment in Animal Models

The effect of ROSI on bone metabolism has been studied *in vivo* in animals to verify possible adverse effects.

The examination of the *in vivo* effect of ROSI administration on the skeletons of adult (6 months old) male nondiabetic C57BL/6 mice [48] showed decrease in total body bone mineral density. Analysis by micro-CT demonstrated a significant loss of bone and changes in several morphometric parameters of bone microarchitecture. Histomorphometric analysis of the trabecular bone of the proximal tibia revealed increase of fat content and number of adipocytes, accompanied by a decrease in osteoblast surface and/or their activity. These features were associated with decreased mRNAs for osteoblast-specific transcription factors *Dlx5* and *Runx2* and the osteoblast-specific marker *Col1A1*. In contrast the mRNA for the adipocyte-specific fatty acids binding protein *aP2* was increased.

Another article described the effect of treatment of adult (5 months old) male and female mice with rosiglitazone, providing other informations on the mechanism of bone loss [49]. A decrease in vertebral bone mineral density was accompanied by increased marrow fat in both vertebral and femoral bone. An increased number of adipocytes and concomitant reduced number of osteoblasts were observed. When mesenchymal stem cells (MSCs) from mice were studied, no difference in MSC number was found when ROSI treated mice were compared with untreated. ROSI had an inhibitory effect on osteoblastogenesis either when added in early MSC cultures, that is, early multipotent progenitors, or when added some days later, that is, when MSC had formed colonies with mixed adipocyte and osteoblast differentiated cells. In this latter case, when MSCs had become later bipotent precursors, ROSI induced a displacement from osteogenesis to adipogenesis. In further later differentiated stages or in mature osteoblasts ROSI had no effect.

Morphologic changes in bone and cell differentiative potential were accompanied by changes in markers specific of bone development, as *Runx2*, *Osterix*, *Col1A1*, and osteocalcin, in favor of markers specific of adipogenesis.

### 3.5. Rosiglitazone: Effect of Treatment in Humans

ROSI is approved and currently utilized as antidiabetic drug in patients affected by type 2 diabetes. Data on efficacy and adverse events are available in large studies in which, besides evaluating glycemic control, the most frequent adverse events are reported: risk of cardiovascular events, weight gain, fluid retention and bone fracture. We focus especially on the risk of bone fractures since other adverse events are more connected with diabetes complications while the effect on bone metabolism has a direct relevance to FOP disease.

A large comparative study in which participated 500 centers in North America, Canada, and Europe, known as A Diabetes Outcome Progression Trial (ADOPT), involved an initial group of 4360 patients [50]. This study was designed to compare monotherapy with ROSI with therapy with metformin or glyburide.

The first published results of ADOPT [51] reported comparable efficacy for the three drugs in term of control of glycemia, with greater durability of glycemic control in ROSI treated patients. Expected adverse events were described: in particular for ROSI, weight gain, increased LDL cholesterol, increased frequency of edema, reduction of hematocrit and C reactive protein level. The authors reported as unexpected a higher rate of fractures in females and not in males (humerus, hand, foot), in the group receiving rosiglitazone compared to metformin and glyburide [51]. A specific analysis of increased risk of fractures within the ADOPT study reported that the difference among the three groups of treatment was evident after one year and that fractures occurred in pre- and postmenopause women in limbs, not in hips or vertebrae [52]. Similar data were obtained by another clinical multicentric study, known as Rosiglitazone evaluated for cardiovascular outcomes in oral agent combination therapy for type 2 Diabetes (RECORD), designed to monitor cardiovascular adverse events. Increased risk of fractures, especially in women, limited to upper and lower limb distal parts, was described [53].

In a more recent study, the effect of Pioglitazone and ROSI was evaluated in a group of 1819 male and female over 40 years old patients who underwent bone fracture and TZD drug exposure [54]. The conclusion was that the risk of fracture was significantly higher during periods of exposure to thiazolidinediones (both rosiglitazone and pioglitazone) compared with unexposed periods. This risk was proportional to duration of treatment and, contrary to the previously cited articles, equal for females and males. 

A confirmation of risk of bone fracture in patients treated with TZD drugs is reported in a recently published article describing results of a cross-sectional study [55].

Another article by the group of authors who participated in the ADOPT study, described results of measures of bone biomarkers. Although not providing conclusive explanation for the clinical findings, it would suggest an effect of TZD drugs primarily on increased bone resorption [56]. The same authors state that further long-term studies will be necessary to understand the mechanisms responsible for TZD-related bone fractures and the consequent possible preventive intervention to limit or abolish this adverse event.

## 4. Conclusions

FOP is one of the most disabling disorders of heterotopic ossification, considered as a “stem-cell disease” in which progenitor cells harbouring a mutated *ACVR1/ALK2* gene, undergo a process of endochondral ossification in response to a mandatory inflammatory trigger. The identity of such cells is still not completely understood and it is likely that both resident progenitor cells of endothelial origin and circulating hematopoietic stem-cells contribute to the heterotopic bone formation [57, 58]. 

Keeping in mind these aspects of FOP pathogenesis, the biological rationale of the use of ROSI in the treatment of FOP is mainly based on the potent inducing effect of ROSI and, in general, TZD drugs, on the PPAR-*γ* transcription factor.

The anti-inflammatory properties of PPAR-*γ* can significantly counteract the inflammatory stimuli responsible for acute phases of the disease preliminary to ossification episodes. Moreover, a relevant PPAR-*γ* effect on bone differentiation and metabolism, based on the ability to trigger and switch differentiation of progenitor cells towards adipogenesis at the expense of osteogenesis, and possibly on mechanisms involving osteoclast-mediated bone resorbtion, is also taking place.

Whether rosiglitazone, due to both anti-inflammatory effects and effects on ossification pathways, can be a better treatment than commonly used glucocorticoids or other anti-inflammatory drugs might be deduced from results of carefully designed clinical experimentation that should take into account potential benefit and undesired effects. ROSI is a well known antidiabetic drug for which a number of adverse effects have been widely described in the scientific literature. Therefore, these have to be carefully considered first in term of awareness and then in term of ability to limit and control such adverse events when proposing ROSI as a potential treatment of FOP.
